# Crystal structure and Hirshfeld surface analysis of 4,5-di­bromo-2-(4-meth­oxy­phen­yl)-2,3,4,4a,5,6,7,7a-octa­hydro-1*H*-4,6-ep­oxy-1*H*-cyclo­penta­[*c*]pyridin-1-one

**DOI:** 10.1107/S2056989021003273

**Published:** 2021-04-16

**Authors:** Dmitriy F. Mertsalov, Nataliya S. Surina, Elena A. Sorokina, Sevim Türktekin Çelikesir, Mehmet Akkurt, Mikhail S. Grigoriev, Sixberth Mlowe

**Affiliations:** a Department of Organic Chemistry, Peoples’ Friendship University of Russia (RUDN University), 6 Miklukho-Maklaya St., 117198, Moscow, Russian Federation; b Department of Physics, Faculty of Sciences, Erciyes University, 38039 Kayseri, Turkey; c Frumkin Institute of Physical Chemistry and Electrochemistry, Russian Academy of Sciences, Leninskiy prospect 31-4, Moscow 119071, Russian Federation; d University of Dar es Salaam, Dar es Salaam University College of Education, Department of Chemistry, PO Box 2329, Dar es Salaam, Tanzania

**Keywords:** crystal structure, ep­oxy­iso­indole group, hydrogen bond, halogen bond, non-covalent inter­actions, Hirshfeld surface analysis

## Abstract

The mol­ecule of the title compound, C_15_H_15_Br_2_NO_3_, comprises a fused tricyclic system consisting of two five-membered rings (cyclo­pentane and tetra­hydro­furan) and one six-membered ring (tetra­hydro­pyridinone). Both five-membered rings of the tricyclic system have envelope conformations, and the conformation of the six-membered cycle is inter­mediate between chair and half-chair. In the crystal, the mol­ecules are linked by C—H⋯O hydrogen bonds and C—H⋯π, C—Br⋯π and C⋯O inter­actions into double layers. The layers are connected into a three-dimensional network by van der Waals inter­actions.

## Abstract 2

The title mol­ecule comprises a fused tricyclic system containing two five-membered rings (cyclo­pentane and tetra­hydro­furan) and one six-membered ring (tetra­hydro­pyridinone). In the crystal, mol­ecules are linked by inter­molecular C—H⋯O hydrogen bonds and C—H⋯π, C—Br⋯π and C⋯O inter­actions into double layers.

## Chemical context   

Iso­indoles are important structural units of many natural products and are widely used as drugs and as building blocks for the construction of new *N*-containing heterocyclic compounds or functional materials (Nadirova *et al.*, 2019 ▸; Zubkov *et al.*, 2011 ▸, 2014 ▸, 2018 ▸). The biological and physical properties of iso­indoles depend on the attached functional groups (Krishna *et al.*, 2021 ▸; Zaytsev *et al.*, 2017 ▸, 2019 ▸, 2020 ▸). Thus, the functionalization of iso­indole moieties at the donor/acceptor sites for non-covalent bonding can improve their biological and photophysical properties as well as their coordination ability (Wicholas *et al.*, 2006 ▸).

On the other hand, non-covalent inter­actions, such as hydrogen, aerogen, halogen, chalcogen, pnictogen, tetrel and icosa­gen bonds, as well as *n*–π*, *π–π* stacking, *π*–cation, *π*–anion, hydro­phobic inter­actions, among others, have recently also attracted much attention and have been demonstrated to play a prominent role in synthesis, catalysis, supra­molecular chemistry, mol­ecular recognition, biological systems, functional materials, *etc*. (Asadov *et al.*, 2016 ▸; Gurbanov *et al.*, 2017 ▸, 2018 ▸; Karmakar *et al.*, 2017 ▸; Kopylovich *et al.*, 2011 ▸; Ma *et al.*, 2017*a* ▸,*b* ▸; 2020 ▸; Mahmudov *et al.*, 2010 ▸, 2012 ▸, 2013 ▸, 2019 ▸, 2020 ▸; Mizar *et al.*, 2012 ▸; Sutradhar *et al.*, 2015 ▸, 2016 ▸). Halogen bonding is a rather spread phenomenon since halogen atoms or ions can form short non-bonding contacts with electron acceptors, electron donors or be inter­connected due to anisotropic charge distribution in halogen atoms (Afkhami *et al.*, 2017 ▸; Maharramov *et al.*, 2018 ▸; Mahmoudi *et al.*, 2017 ▸, 2019 ▸; Shixaliyev *et al.*, 2014 ▸). In fact, functionalization of iso­indoles with donor or acceptor sites for non-covalent bonding greatly affects their supra­molecular arrangements (Gurbanov *et al.*, 2021 ▸).
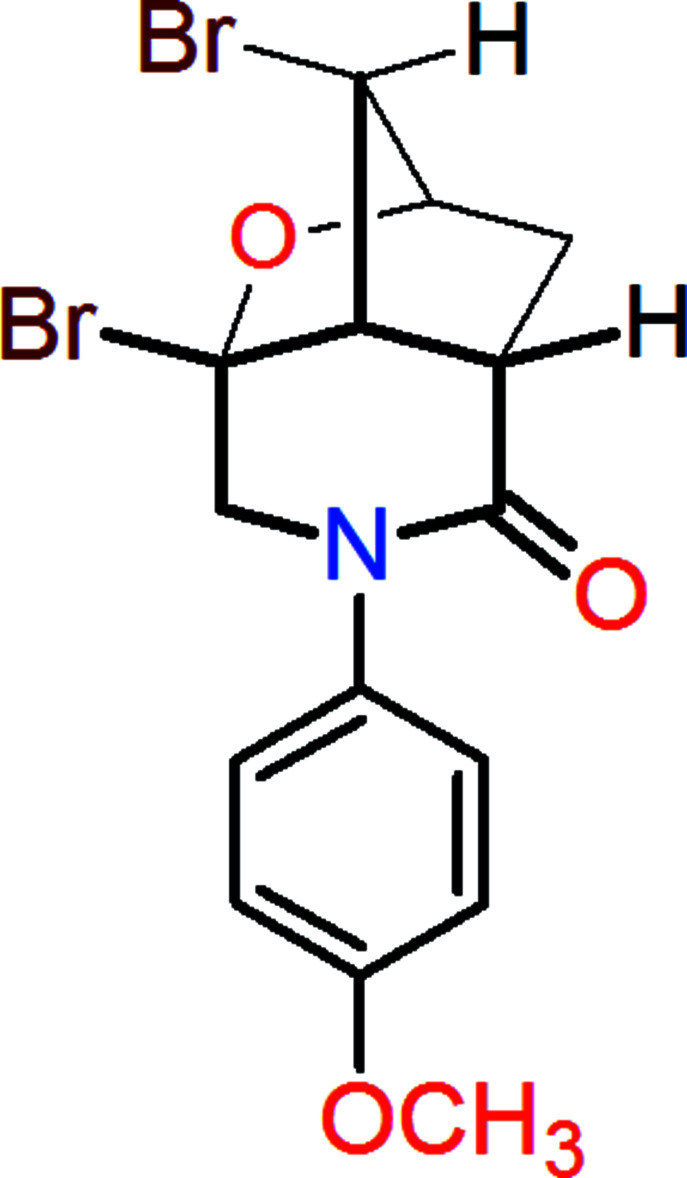


**Figure 1:**
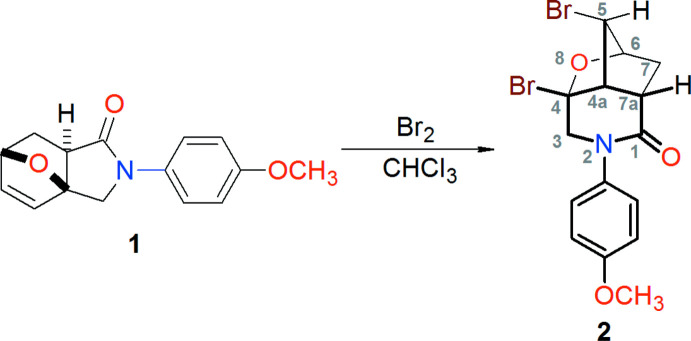
Synthesis of the title compound (**2**).

In a continuation of our work in this direction, we have functionalized a new iso­indole (**1**) (Zaytsev *et al.*, 2020 ▸) by reaction with bromine yielding 4,5-di­bromo-2-(4-meth­oxy­phen­yl)-2,3,4,4a,5,6,7,7a-octa­hydro-1*H*-4,6-ep­oxy-1*H*-cyclo­penta­[*c*]pyridin-1-one (**2**; Fig. 1 ▸), which provides examples of C—Br⋯O halogen bonds as well as of C—H⋯O and C—H⋯π types of inter­molecular hydrogen bonds.

## Structural commentary   

**Figure 2:**
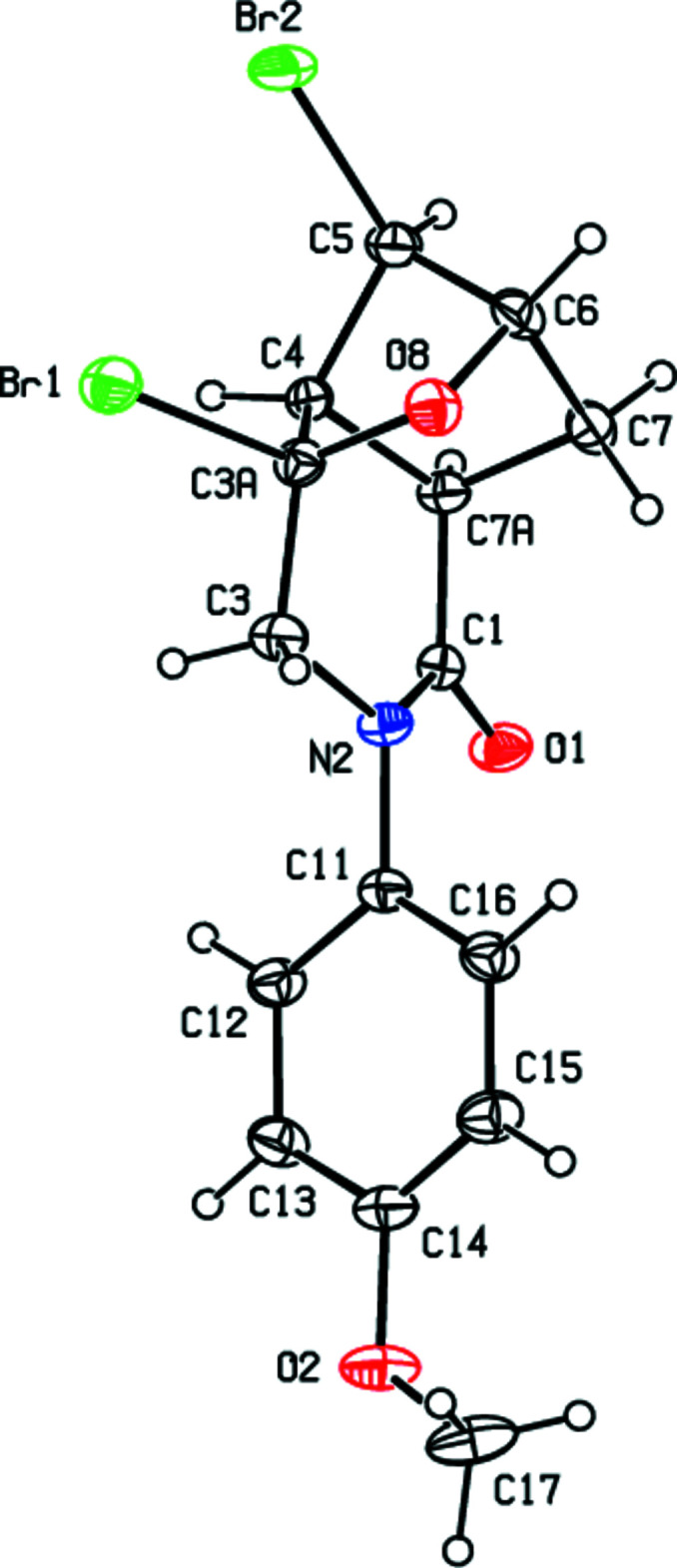
The mol­ecular structure of **2**, with displacement ellipsoids for non-hydrogen atoms drawn at the 30% probability level.

As shown in Fig. 2 ▸, the mol­ecule of the title compound, **2**, comprises a fused tricyclic system containing two five-membered rings (cyclo­pentane C4–C7/C7*A* and tetra­hydro­furan C3*A*/C4–C6/O8) and one six-membered ring (tetra­hydro­pyridinone C1/N2/C3/C3*A*/C4/C7*A*). Both five-membered rings of the tricyclic fragment have envelope conformations with the C5 atom as the flap, and the six-membered ring adopts a flattened chair conformation with the N2 and C4 atoms displaced by 0.276 (3) and −0.670 (4) Å, respectively, from the mean plane through the remaining four atoms. The environment of atom N2, being close to trigonal–planar, is slightly pyramidalized due to steric reasons [the sum of bond angles at N2 is 356.7 (5)°]. The dihedral angle between the mean planes of the tetra­hydro­pyridinone and benzene rings is 82.82 (16)°.

## Supra­molecular features and Hirshfeld surface analysis   

**Figure 3:**
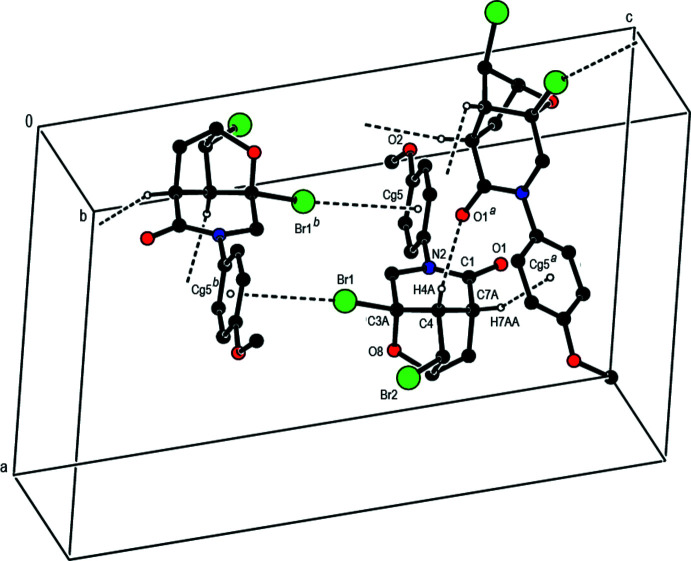
Inter­molecular C—H⋯O, C—H⋯π and C—Br⋯π inter­actions [symmetry codes: (*a*) 1 − *x*, −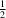 + *y*, 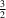 − *z*; (*b*) 1 − *x*, −*y*, 1 − *z*].

In the crystal, mol­ecules are linked by inter­molecular C—H⋯O hydrogen bonds, C—H⋯π, C—Br⋯π and C⋯O inter­actions into double layers parallel to (001) (Tables 1 ▸ and 2 ▸; Figs. 3 ▸, 4 ▸ and 5 ▸). The layers are further connected into a three-dimensional network by van der Waals inter­actions.

**Figure 6:**
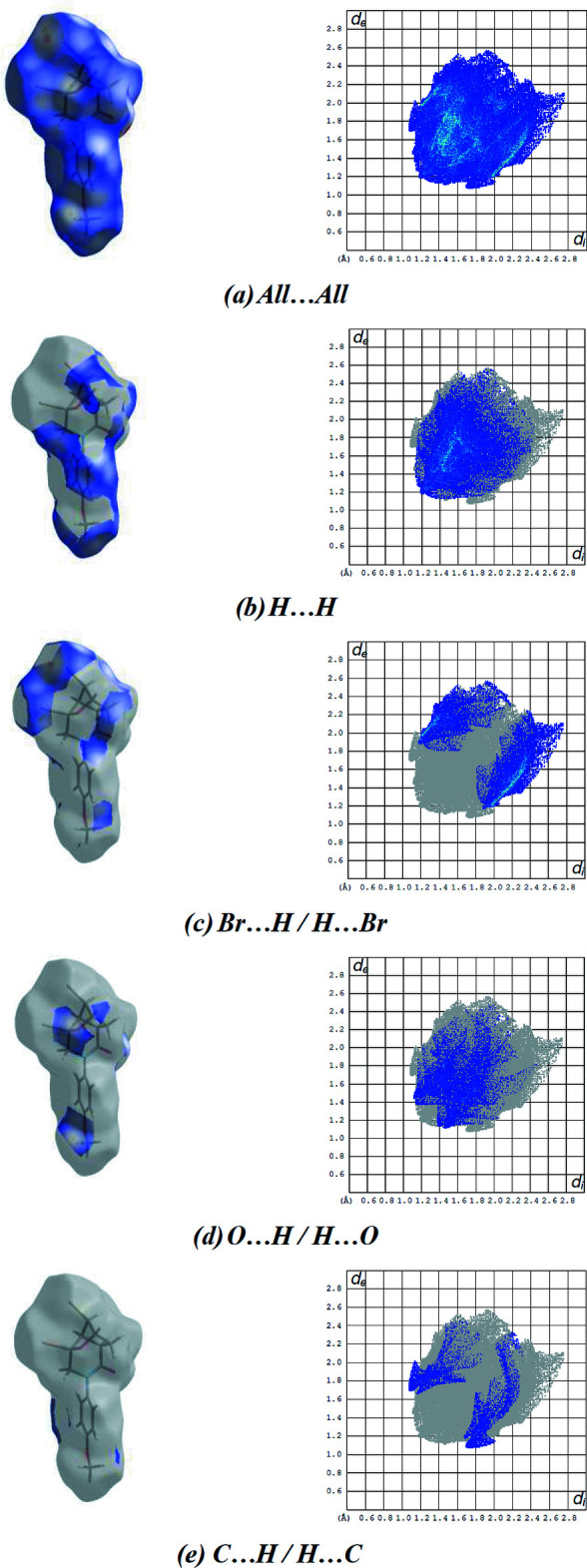
A view of the three-dimensional Hirshfeld surfaces and the two-dimensional fingerprint plots for **2**, showing (*a*) all inter­actions and delineated into (*b*) H⋯H, (*c*) Br⋯H/H⋯Br, (*d*) O⋯H/H⋯O and (*e*) C⋯H/H⋯C inter­actions. The *d*_i_ and *d*_e_ values are the closest inter­nal and external distances (Å) from given points on the Hirshfeld surface.

The Hirshfeld surface for **2** mapped over *d*_norm_ and the associated two-dimensional fingerprint plots are shown in Fig. 6 ▸. All of them were generated using *CrystalExplorer17* (Turner *et al.*, 2017 ▸). Red spots on the Hirshfeld surface mapped over *d*_norm_ in the colour range −0.2469 to 1.1913 a.u. confirm the inter­molecular contacts (Tables 1 ▸ and 2 ▸). The fingerprint plots are given for all contacts and those delineated into H⋯H (41.1%; Fig. 6 ▸ *b*), Br⋯H/H⋯Br (24.5%; Fig. 6 ▸ *c*), O⋯H/H⋯O (16.9%; Fig. 6 ▸ *d*) and C⋯H/H⋯C (8.2%; Fig. 6 ▸ *e*) contacts. All contributions to the Hirshfeld surface are given in Table 3 ▸. The large number of H⋯H, Br⋯H/H⋯Br and O⋯H/H⋯O inter­actions suggest that van der Waals inter­actions and hydrogen bonding play the major roles in the crystal packing (Hathwar *et al.*, 2015 ▸).

## Database survey   

A survey of the Cambridge Structural Database (CSD version 5.41, update of March 2020; Groom *et al.*, 2016 ▸) reveals two compounds containing the octa­hydro-1*H*-4,6-ep­oxy­cyclo­penta­[c]pyridin-1-one skeleton, *viz*. methyl *rac*-(1S*,3R*,7R*,8R*,9R*,10S*)-3,9-diacet­oxy-6-oxo-5-phenyl-2-oxa-5-aza­tri­cyclo­[5.2.1.0^3,8^]decane-10-carboxyl­ate ethanol solvate (refcode RUJJUC; Gurbanov *et al.*, 2009 ▸) and methyl *rac*-(1S*,2R*,4R*,13S*,14R*,15R*,16S*,117S*)-1,16-diacet­oxy-12-oxo-4-(2-oxopyrrolidin-1-yl)-18-oxa-11-aza­penta­cyclo­[13.2.1.0^2,11^.0^5,10^.0^13,17^]octa­deca-5,7,9-triene-14-carboxyl­ate sesquihydrate (HUGJUP; Gurbanov *et al.*, 2010 ▸).

The racemic crystal of RUJJUC consists of enanti­omeric pairs with the configurations *rac*-4R*,4aR*,5R*,6S*,7S*,7aR*. The ethanol solvate mol­ecule is bound to the mol­ecule of RUJJUC by a strong O—H⋯O hydrogen bond. In the crystal of HUGJUP, there are three O—H⋯O hydrogen bonds, which link the organic mol­ecules and water mol­ecules into layers parallel to (001). The layers are further linked into a three-dimensional framework by attractive inter­molecular carbon­yl–carbonyl inter­actions.

## Synthesis and crystallization   

A solution of isoindolone **1** (1.2 mmol) and bromine (1.75 mmol) in dry chloro­form (3 mL) was stirred for 5 h (TLC control, EtOAc–hexane, 1:1). The reaction mixture was poured into H_2_O (30 mL) and extracted with CHCl_3_ (3 × 20 mL). The combined extracts were dried over anhydrous Na_2_SO_4_ and concentrated *in vacuo*. The obtained solid was recrystallized by slow evaporation from EtOH to give single crystals of dibromide **2** suitable for X-ray analysis. Colourless needles, yield 0.42 g (86%). M.p. > 478 K (decomposition). IR (KBr), ν (cm^−1^): 1671 (N—C=O), 610 (C—Br). ^1^H NMR (DMSO-*d*_6_, 700.1 MHz, 298 K): δ = 7.19 (*d*, 2H, H2, H6 H-Ph, *J* = 8.8), 6.94 (*d*, 2H, H3, H5 H-Ph, *J* = 8.8), 4.92 (*s*, 1H, H-6), 4.62 (*d*, 1H, *J* = 13.1), 4.11 (*d*, 1H, H-3, *J* = 13.1), 4.61 (*d*, 1H, H-5), 3.57 (*s*, 3H, OCH_3_), 3.67 (*d*, 1H, H-4a, *J* = 3.9), 2.93 (*dt*, 1H, H-7a, *J* = 3.9, *J* = 11.9), 2.41 (*t*, 1H, H-7exo, *J* = 11.9), 1.53 (*dd*, 1H, H-7endo, *J* = 3.9, *J* = 11.9). ^13^C NMR (DMSO-*d*_6_, 176.0 MHz, 298 K): *δ* = 169.7, 157.8, 133.7, 127.4 (2C), 114.1 (2C), 99.3, 86.0, 61.0, 55.3, 51.7, 48.5, 39.0, 36.4. MS (APCI): *m*/*z* = 420 [*M* + H]^+^ (^81^Br), 418 [*M* + H]^+^ (^81^Br, ^79^Br), 416 [*M* + H]^+^ (^79^Br).

## Refinement   

Crystal data, data collection and structure refinement details are summarized in Table 4 ▸. All H atoms were positioned geometrically (C—H = 0.93 − 0.98 Å) and refined using a riding model, with *U*_iso_(H) = 1.2*U*_eq_(C) or 1.5*U*_eq_(C-meth­yl). Owing to poor agreement between observed and calculated intensities, four outliers (6 0 2, 1 2 0, 0 3 3 and 0 0 6) were omitted from the final cycles of refinement.

## Supplementary Material

Crystal structure: contains datablock(s) I. DOI: 10.1107/S2056989021003273/yk2149sup1.cif

Structure factors: contains datablock(s) I. DOI: 10.1107/S2056989021003273/yk2149Isup2.hkl

Supporting information file. DOI: 10.1107/S2056989021003273/yk2149Isup3.cml

CCDC reference: 2053095

Additional supporting information:  crystallographic information; 3D view; checkCIF report

## Figures and Tables

**Figure 4:**
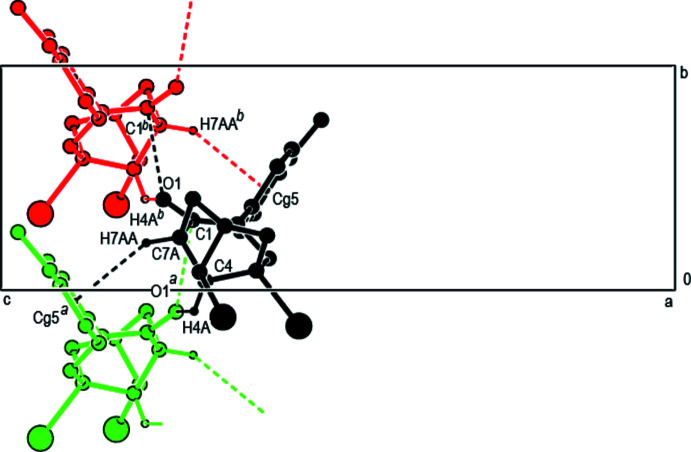
A fragment of the double layer in **2** formed by inter­molecular C—H⋯O, C⋯O and C—H⋯π contacts [symmetry codes: (*a*) 1 − *x*, −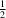 + *y*, 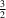 − *z*; (*b*) 1 − *x*, 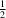 + *y*, 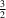 − *z*].

**Figure 5:**
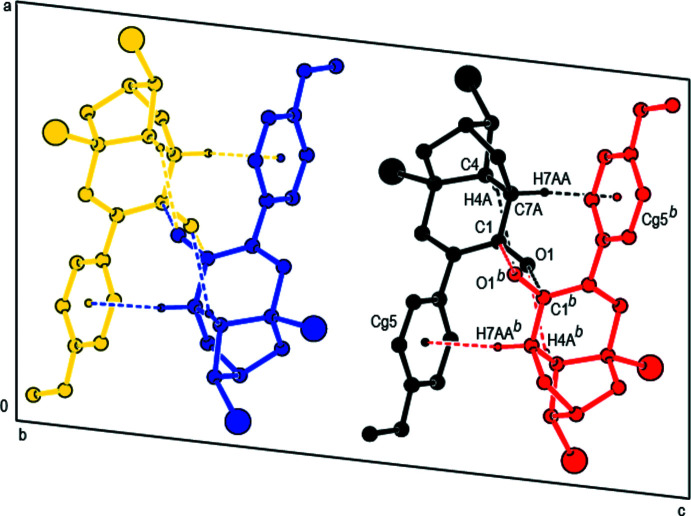
Packing diagram of **2** viewed along the *b*-axis direction showing the inter­molecular C—H⋯O and C—H⋯π contacts [symmetry code: (*b*) 1 − *x*, 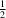 + *y*, 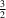 − *z*].

**Table 1:** Hydrogen-bond geometry (Å, °) *Cg* is the centroid of the C11–C16 ring.

*D*—H⋯*A*	*D*—H	H⋯*A*	*D*⋯*A*	*D*—H⋯*A*
C4—H4*A*⋯O1^i^	0.98	2.57	3.092 (3)	114
C7*A*—H7*AA*⋯*Cg*5^i^	0.98	2.69	3.573 (4)	150

Symmetry code: (i) -x+1, y-{\script{1\over 2}}, -z+{\script{3\over 2}}.

**Table 2:** Summary of shortest van der Waals contacts (Å) in the title compound

Contact	Distance	Symmetry operation
Br1⋯*Cg*5	3.7132 (15)	1 − *x*, −*y*, 1 − *z*
Br2⋯O2	3.316 (3)	1 + *x*, −1 + *y*, *z*
Br2⋯H17*A*	3.13	1 + *x*, −1 + *y*, *z*
Br2⋯H5*A*	3.13	2 − *x*, −{1\over 2} + *y*, {3\over 2} − *z*
O1⋯C1	2.822 (4)	1 − *x*, {1\over 2} + *y*, {3\over 2} − *z*
O8⋯H17*C*	2.66	1 − *x*, 1 − *y*, 1 − *z*
H4*A*⋯H7*A*	2.54	*x*, −1 + *y*, *z*
H7*B*⋯H12*A*	2.55	1 − *x*, {1\over 2} + *y*, {3\over 2} − *z*

**Table 3:** Percentage contributions of inter­atomic contacts to the Hirshfeld surface for the title compound

Contact	Percentage contribution
H⋯H	41.1
Br⋯H/H⋯Br	24.5
O⋯H/H⋯O	16.9
C⋯H/H⋯C	8.2
Br⋯C/C⋯Br	4.3
Br⋯O/O⋯Br	2.6
O⋯C/C⋯O	1.5
O⋯O	0.8
O⋯N/N⋯O	0.1

**Table 4:** Experimental details

Crystal data	
Chemical formula	C_15_H_15_Br_2_NO_3_
*M*_r_	417.10
Crystal system, space group	Monoclinic, *P*2_1_/*c*
Temperature (K)	296
*a*, *b*, *c* (Å)	12.0238 (12), 6.4316 (7), 19.463 (2)
β (°)	96.618 (4)
*V* (Å^3^)	1495.1 (3)
*Z*	4
Radiation type	Mo *K*α
μ (mm^−1^)	5.43
Crystal size (mm)	0.40 × 0.12 × 0.06

Data collection	
Diffractometer	Bruker Kappa APEXII area-detector diffractometer
Absorption correction	Multi-scan (*SADABS*; Bruker, 2013 ▸)
*T*_min_, *T*_max_	0.461, 0.736
No. of measured, independent and observed [*I* > 2σ(*I*)] reflections	13519, 3429, 2522
*R*_int_	0.043
(sin θ/λ)_max_ (Å^−1^)	0.650

Refinement	
*R*[*F* ^2^ > 2σ(*F* ^2^)], *wR*(*F* ^2^), *S*	0.037, 0.091, 1.01
No. of reflections	3429
No. of parameters	190
H-atom treatment	H-atom parameters constrained
Δρ_max_, Δρ_min_ (e Å^−3^)	1.30, −0.62

Computer programs: *APEX2* and *SAINT* (Bruker, 2013 ▸), *SHELXT* (Sheldrick, 2015*a* ▸), *SHELXL* (Sheldrick, 2015*b* ▸), *ORTEP-3 for Windows* (Farrugia, 2012 ▸) and *PLATON* (Spek, 2020 ▸).

## supplementary crystallographic information

### Crystal data

**Table:**

C_15_H_15_Br_2_NO_3_	*F*(000) = 824
*M**_r_* = 417.10	*D*_x_ = 1.853 Mg m^−^^3^
Monoclinic, *P*2_1_/*c*	Mo *K*α radiation, λ = 0.71073 Å
*a* = 12.0238 (12) Å	Cell parameters from 2677 reflections
*b* = 6.4316 (7) Å	θ = 2.9–24.4°
*c* = 19.463 (2) Å	µ = 5.43 mm^−^^1^
β = 96.618 (4)°	*T* = 296 K
*V* = 1495.1 (3) Å^3^	Needle, colourless
*Z* = 4	0.40 × 0.12 × 0.06 mm

### Data collection

**Table:**

Bruker Kappa APEXII area-detector diffractometer	2522 reflections with *I* > 2σ(*I*)
φ and ω scans	*R*_int_ = 0.043
Absorption correction: multi-scan (SADABS; Bruker, 2013)	θ_max_ = 27.5°, θ_min_ = 3.4°
*T*_min_ = 0.461, *T*_max_ = 0.736	*h* = −15→15
13519 measured reflections	*k* = −8→8
3429 independent reflections	*l* = −24→25

### Refinement

**Table:**

Refinement on *F*^2^	0 restraints
Least-squares matrix: full	Hydrogen site location: inferred from neighbouring sites
*R*[*F*^2^ > 2σ(*F*^2^)] = 0.037	H-atom parameters constrained
*wR*(*F*^2^) = 0.091	*w* = 1/[σ^2^(*F*_o_^2^) + (0.0431*P*)^2^ + 0.868*P*] where *P* = (*F*_o_^2^ + 2*F*_c_^2^)/3
*S* = 1.01	(Δ/σ)_max_ = 0.001
3429 reflections	Δρ_max_ = 1.30 e Å^−^^3^
190 parameters	Δρ_min_ = −0.62 e Å^−^^3^

### Special details

**Table:**

Geometry. All esds (except the esd in the dihedral angle between two l.s. planes) are estimated using the full covariance matrix. The cell esds are taken into account individually in the estimation of esds in distances, angles and torsion angles; correlations between esds in cell parameters are only used when they are defined by crystal symmetry. An approximate (isotropic) treatment of cell esds is used for estimating esds involving l.s. planes.

### Fractional atomic coordinates and isotropic or equivalent isotropic displacement parameters (Å^2^)

**Table:**

	*x*	*y*	*z*	*U*_iso_*/*U*_eq_	
Br1	0.69848 (3)	−0.16042 (6)	0.55820 (2)	0.04500 (13)	
Br2	0.94126 (3)	−0.12067 (7)	0.67093 (2)	0.04941 (14)	
O1	0.51267 (19)	0.4036 (4)	0.75946 (12)	0.0386 (6)	
O2	0.0858 (2)	0.5884 (5)	0.57203 (14)	0.0504 (7)	
O8	0.75480 (19)	0.2430 (4)	0.60541 (12)	0.0349 (5)	
N2	0.5178 (2)	0.2933 (4)	0.64953 (13)	0.0284 (6)	
C1	0.5603 (3)	0.3115 (5)	0.71658 (17)	0.0273 (7)	
C3A	0.6814 (3)	0.0868 (5)	0.62134 (16)	0.0289 (7)	
C3	0.5590 (3)	0.1411 (5)	0.60289 (17)	0.0323 (8)	
H3A	0.548416	0.195612	0.556131	0.039*	
H3B	0.514694	0.015166	0.603693	0.039*	
C4	0.7156 (2)	0.0398 (5)	0.69685 (16)	0.0257 (6)	
H4A	0.691323	−0.094568	0.713661	0.031*	
C5	0.8414 (3)	0.0806 (5)	0.70565 (18)	0.0336 (8)	
H5A	0.865966	0.106512	0.754666	0.040*	
C6	0.8301 (3)	0.2855 (6)	0.66820 (19)	0.0358 (8)	
H6A	0.901320	0.350442	0.660335	0.043*	
C7A	0.6785 (3)	0.2352 (5)	0.73476 (17)	0.0279 (7)	
H7AA	0.691219	0.210486	0.784733	0.033*	
C7	0.7624 (3)	0.4062 (5)	0.71548 (19)	0.0355 (8)	
H7A	0.723577	0.522331	0.691645	0.043*	
H7B	0.808923	0.456851	0.756063	0.043*	
C11	0.4066 (3)	0.3716 (5)	0.62824 (16)	0.0280 (7)	
C12	0.3137 (3)	0.2636 (6)	0.64507 (18)	0.0342 (8)	
H12A	0.322793	0.139531	0.669624	0.041*	
C13	0.2078 (3)	0.3407 (6)	0.62528 (19)	0.0392 (8)	
H13A	0.145407	0.270187	0.637376	0.047*	
C14	0.1944 (3)	0.5227 (6)	0.58748 (17)	0.0351 (8)	
C15	0.2861 (3)	0.6274 (6)	0.56874 (19)	0.0401 (9)	
H15A	0.276873	0.747622	0.542168	0.048*	
C16	0.3926 (3)	0.5508 (6)	0.59010 (18)	0.0362 (8)	
H16A	0.455034	0.621952	0.578373	0.043*	
C17	0.0658 (4)	0.7577 (8)	0.5251 (2)	0.0597 (12)	
H17A	−0.012785	0.788516	0.518630	0.090*	
H17B	0.106390	0.877628	0.543611	0.090*	
H17C	0.090453	0.721139	0.481502	0.090*	

### Atomic displacement parameters (Å^2^)

**Table:**

	*U*^11^	*U*^22^	*U*^33^	*U*^12^	*U*^13^	*U*^23^
Br1	0.0406 (2)	0.0497 (2)	0.0443 (2)	0.00670 (17)	0.00301 (16)	−0.01833 (18)
Br2	0.0286 (2)	0.0543 (3)	0.0653 (3)	0.01404 (17)	0.00518 (17)	−0.00677 (19)
O1	0.0321 (13)	0.0467 (14)	0.0370 (14)	0.0102 (11)	0.0042 (11)	−0.0118 (11)
O2	0.0300 (14)	0.0703 (19)	0.0501 (16)	0.0171 (13)	0.0013 (12)	0.0129 (14)
O8	0.0350 (13)	0.0375 (13)	0.0338 (13)	−0.0033 (11)	0.0102 (10)	0.0049 (10)
N2	0.0242 (14)	0.0339 (15)	0.0275 (14)	0.0068 (11)	0.0046 (11)	−0.0004 (11)
C1	0.0246 (16)	0.0260 (16)	0.0311 (17)	0.0002 (12)	0.0026 (13)	−0.0017 (13)
C3A	0.0286 (17)	0.0312 (17)	0.0276 (17)	0.0029 (14)	0.0058 (13)	−0.0043 (13)
C3	0.0279 (17)	0.042 (2)	0.0268 (17)	0.0045 (14)	0.0016 (13)	−0.0055 (14)
C4	0.0231 (16)	0.0246 (15)	0.0297 (16)	0.0016 (12)	0.0040 (12)	0.0018 (13)
C5	0.0240 (17)	0.0400 (19)	0.0364 (19)	0.0043 (14)	0.0017 (14)	−0.0062 (15)
C6	0.0236 (17)	0.0400 (19)	0.045 (2)	−0.0066 (14)	0.0069 (15)	−0.0020 (16)
C7A	0.0233 (16)	0.0329 (17)	0.0274 (16)	0.0052 (13)	0.0024 (13)	−0.0015 (13)
C7	0.0302 (18)	0.0354 (18)	0.041 (2)	−0.0062 (15)	0.0031 (15)	−0.0056 (15)
C11	0.0240 (16)	0.0323 (17)	0.0271 (16)	0.0041 (13)	0.0008 (13)	0.0003 (13)
C12	0.0299 (18)	0.0363 (19)	0.0368 (19)	0.0046 (15)	0.0051 (14)	0.0077 (15)
C13	0.0274 (18)	0.046 (2)	0.044 (2)	−0.0011 (16)	0.0053 (15)	0.0030 (17)
C14	0.0276 (18)	0.048 (2)	0.0287 (17)	0.0113 (15)	0.0003 (14)	−0.0031 (15)
C15	0.038 (2)	0.045 (2)	0.038 (2)	0.0084 (16)	0.0045 (16)	0.0124 (16)
C16	0.0285 (18)	0.039 (2)	0.041 (2)	−0.0003 (15)	0.0060 (15)	0.0107 (16)
C17	0.048 (2)	0.081 (3)	0.050 (3)	0.035 (2)	0.004 (2)	0.018 (2)

### Geometric parameters (Å, º)

**Table:**

Br1—C3A	2.034 (3)	C6—C7	1.512 (5)
Br2—C5	1.940 (3)	C6—H6A	0.9800
O1—C1	1.219 (4)	C7A—C7	1.567 (5)
O2—C14	1.372 (4)	C7A—H7AA	0.9800
O2—C17	1.424 (5)	C7—H7A	0.9700
O8—C3A	1.395 (4)	C7—H7B	0.9700
O8—C6	1.461 (4)	C11—C16	1.371 (4)
N2—C1	1.351 (4)	C11—C12	1.386 (5)
N2—C11	1.443 (4)	C12—C13	1.380 (5)
N2—C3	1.460 (4)	C12—H12A	0.9300
C1—C7A	1.507 (4)	C13—C14	1.382 (5)
C3A—C4	1.510 (4)	C13—H13A	0.9300
C3A—C3	1.516 (4)	C14—C15	1.376 (5)
C3—H3A	0.9700	C15—C16	1.391 (5)
C3—H3B	0.9700	C15—H15A	0.9300
C4—C5	1.525 (4)	C16—H16A	0.9300
C4—C7A	1.549 (4)	C17—H17A	0.9600
C4—H4A	0.9800	C17—H17B	0.9600
C5—C6	1.505 (5)	C17—H17C	0.9600
C5—H5A	0.9800		
			
C14—O2—C17	117.5 (3)	C7—C6—H6A	114.7
C3A—O8—C6	107.2 (2)	C1—C7A—C4	117.9 (3)
C1—N2—C11	118.8 (3)	C1—C7A—C7	109.3 (3)
C1—N2—C3	122.8 (3)	C4—C7A—C7	103.1 (3)
C11—N2—C3	115.1 (3)	C1—C7A—H7AA	108.7
O1—C1—N2	123.3 (3)	C4—C7A—H7AA	108.7
O1—C1—C7A	120.1 (3)	C7—C7A—H7AA	108.7
N2—C1—C7A	116.1 (3)	C6—C7—C7A	101.1 (3)
O8—C3A—C4	104.6 (3)	C6—C7—H7A	111.6
O8—C3A—C3	113.8 (3)	C7A—C7—H7A	111.6
C4—C3A—C3	115.1 (3)	C6—C7—H7B	111.6
O8—C3A—Br1	108.6 (2)	C7A—C7—H7B	111.6
C4—C3A—Br1	113.4 (2)	H7A—C7—H7B	109.4
C3—C3A—Br1	101.5 (2)	C16—C11—C12	119.8 (3)
N2—C3—C3A	113.4 (3)	C16—C11—N2	120.1 (3)
N2—C3—H3A	108.9	C12—C11—N2	120.1 (3)
C3A—C3—H3A	108.9	C13—C12—C11	119.8 (3)
N2—C3—H3B	108.9	C13—C12—H12A	120.1
C3A—C3—H3B	108.9	C11—C12—H12A	120.1
H3A—C3—H3B	107.7	C12—C13—C14	120.0 (3)
C3A—C4—C5	103.3 (3)	C12—C13—H13A	120.0
C3A—C4—C7A	103.9 (2)	C14—C13—H13A	120.0
C5—C4—C7A	98.2 (2)	O2—C14—C15	124.2 (3)
C3A—C4—H4A	116.3	O2—C14—C13	115.3 (3)
C5—C4—H4A	116.3	C15—C14—C13	120.5 (3)
C7A—C4—H4A	116.3	C14—C15—C16	119.1 (3)
C6—C5—C4	93.7 (2)	C14—C15—H15A	120.5
C6—C5—Br2	116.1 (2)	C16—C15—H15A	120.5
C4—C5—Br2	119.5 (2)	C11—C16—C15	120.8 (3)
C6—C5—H5A	108.8	C11—C16—H16A	119.6
C4—C5—H5A	108.8	C15—C16—H16A	119.6
Br2—C5—H5A	108.8	O2—C17—H17A	109.5
O8—C6—C5	104.7 (3)	O2—C17—H17B	109.5
O8—C6—C7	106.2 (3)	H17A—C17—H17B	109.5
C5—C6—C7	100.3 (3)	O2—C17—H17C	109.5
O8—C6—H6A	114.7	H17A—C17—H17C	109.5
C5—C6—H6A	114.7	H17B—C17—H17C	109.5
			
C11—N2—C1—O1	−5.2 (5)	O1—C1—C7A—C4	151.2 (3)
C3—N2—C1—O1	−163.7 (3)	N2—C1—C7A—C4	−36.4 (4)
C11—N2—C1—C7A	−177.4 (3)	O1—C1—C7A—C7	−91.6 (4)
C3—N2—C1—C7A	24.1 (4)	N2—C1—C7A—C7	80.9 (3)
C6—O8—C3A—C4	0.7 (3)	C3A—C4—C7A—C1	50.2 (3)
C6—O8—C3A—C3	127.1 (3)	C5—C4—C7A—C1	156.1 (3)
C6—O8—C3A—Br1	−120.6 (2)	C3A—C4—C7A—C7	−70.3 (3)
C1—N2—C3—C3A	−29.3 (4)	C5—C4—C7A—C7	35.6 (3)
C11—N2—C3—C3A	171.5 (3)	O8—C6—C7—C7A	68.9 (3)
O8—C3A—C3—N2	−73.6 (4)	C5—C6—C7—C7A	−39.9 (3)
C4—C3A—C3—N2	47.1 (4)	C1—C7A—C7—C6	−124.2 (3)
Br1—C3A—C3—N2	169.9 (2)	C4—C7A—C7—C6	2.0 (3)
O8—C3A—C4—C5	−31.7 (3)	C1—N2—C11—C16	106.7 (4)
C3—C3A—C4—C5	−157.3 (3)	C3—N2—C11—C16	−93.2 (4)
Br1—C3A—C4—C5	86.4 (3)	C1—N2—C11—C12	−74.6 (4)
O8—C3A—C4—C7A	70.4 (3)	C3—N2—C11—C12	85.5 (4)
C3—C3A—C4—C7A	−55.3 (3)	C16—C11—C12—C13	−2.1 (5)
Br1—C3A—C4—C7A	−171.5 (2)	N2—C11—C12—C13	179.2 (3)
C3A—C4—C5—C6	47.1 (3)	C11—C12—C13—C14	1.4 (5)
C7A—C4—C5—C6	−59.3 (3)	C17—O2—C14—C15	9.8 (5)
C3A—C4—C5—Br2	−75.8 (3)	C17—O2—C14—C13	−171.3 (4)
C7A—C4—C5—Br2	177.8 (2)	C12—C13—C14—O2	−178.4 (3)
C3A—O8—C6—C5	31.2 (3)	C12—C13—C14—C15	0.6 (5)
C3A—O8—C6—C7	−74.4 (3)	O2—C14—C15—C16	177.0 (3)
C4—C5—C6—O8	−47.4 (3)	C13—C14—C15—C16	−1.9 (6)
Br2—C5—C6—O8	78.1 (3)	C12—C11—C16—C15	0.8 (5)
C4—C5—C6—C7	62.6 (3)	N2—C11—C16—C15	179.5 (3)
Br2—C5—C6—C7	−171.9 (2)	C14—C15—C16—C11	1.2 (6)

### Hydrogen-bond geometry (Å, º)

*Cg* is the centroid of the C11–C16 ring.

**Table:**

*D*—H···*A*	*D*—H	H···*A*	*D*···*A*	*D*—H···*A*
C4—H4*A*···O1^i^	0.98	2.57	3.092 (3)	114
C7*A*—H7*AA*···*Cg*5^i^	0.98	2.69	3.573 (4)	150

Symmetry code: (i) −*x*+1, *y*−1/2, −*z*+3/2.
